# Sericin cream reduces pruritus in hemodialysis patients: a randomized, double-blind, placebo-controlled experimental study

**DOI:** 10.1186/1471-2369-13-119

**Published:** 2012-09-24

**Authors:** Pornanong Aramwit, Orathai Keongamaroon, Tippawan Siritientong, Nipaporn Bang, Ouppatham Supasyndh

**Affiliations:** 1 Bioactive Resources for Innovative Clinical Applications Research Unit and Department of Pharmacy Practice, Faculty of Pharmaceutical Sciences, Chulalongkorn University, Bangkok 10330, Thailand; 2 Division of Nephrology, Department of Medicine, Phramongkutklao Hospital and College of Medicine, Bangkok, Thailand

**Keywords:** Pruritus, Hemodialysis, Sericin, Hydration, Inflammation, Pigmentation

## Abstract

**Background:**

Uremic pruritus (UP) is a significant complication in ESRD patients and substantially impairs their quality of life. UP is considered to be a skin manifestation of chronic inflammation. Because sericin can suppress the release of pro-inflammatory cytokines, the purpose of this study was to investigate the short-term safety and efficacy of sericin cream for treating UP in hemodialysis patients.

**Methods:**

This study used a double-blind design to investigate the effects of random topical administration of sericin cream and cream base (placebo) on either the right or left extremities of hemodialysis patients for 6 weeks. Skin hydration, irritation and pigmentation were evaluated every 2 weeks using Skin Diagnostic SD27. The visual analog scale for itching was also evaluated every 2 weeks, and the Kidney Disease Quality of Life Short Form was performed on the day of each patient’s enrollment and after 6 weeks of treatment.

**Results:**

Fifty dialysis patients were enrolled, 47 of which completed the study. The hydration of the skin of the patients’ extremities increased significantly after administration of sericin cream; significant differences were found between sericin treatment and control after 6 weeks of treatment (*p* = 0.041 for arms and *p* = 0.022 for legs, respectively). Moreover, a significant difference was also found in skin irritation between the two treatments (*p* = 0.013 for arms and *p* = 0.027 for legs, respectively). At the end of the study, the skin pigmentation level was significantly reduced on both the arms (*p* = 0.032) and legs (*p* = 0.021) of the sericin-treated side compared with the side treated with cream base. The mean itching score decreased significantly from moderate to severe at the time of enrollment to mild pruritus after 6 weeks of treatment (*p* = 0.002). A better quality of life was found in all domains tested although statistically significant differences before and after treatment was found only in the patients’ pain scores, the effect of kidney disease on daily life, sleep quality and symptoms or problems related to kidney disease.

**Conclusions:**

We conclude that sericin cream has a high potential for reducing UP in hemodialysis patients.

The trial registration number of this study is ISRCTN16019033; its public title is “sericin cream reduces pruritus in hemodialysis patients”.

## Background

Itching associated with end-stage renal disease (ESRD) or uremic pruritus (UP) affects between 20 and 50% of renal failure patients [1-3] who have no primary skin disease or systemic or psychological dysfunction that might cause pruritus. In addition, approximately 80% of patients undergoing hemodialysis were found to be affected by UP [4-6]. Unfortunately, dialysis has only a slight impact on pruritus [7].

Pruritus is an unpleasant symptom that negatively impacts a patient’s quality of life. The impact of moderate/severe uremic pruritus on the mortality of patients with ESRD seems to be associated with sleep disturbance rather than with uremic pruritus *per se*[8,9]. Moreover, in the recent Dialysis Outcomes and Practice Patterns Study II (DOPPS II) trial, pruritus was associated with depression, sleep disturbance and increased mortality risk [8,10]. The pathophysiology of UP is still unclear; many hypotheses have been proposed to explain its occurrence, including xerosis and hypohidrosis (a condition in which the skin is usually atrophic and dry), the presence of pruritogenic cytokines (histamine, kallikrein, interleukin [IL]-2, acetylcholine and other substances that are released by histamine-mediated mast cell stimulation and may lower the UP threshold), secondary hyperparathyroidism, immune-inflammatory reactions in which sericin cream is thought to play a central role in producing an anti-inflammatory effect, the uremic neuropathy hypothesis and the opioid hypothesis (k-opioid receptor understimulation and overexpression of μ-opioid receptors) [7,8].

Sericin, a biopolymer with a high molecular weight, is a water-soluble protein that is obtained from the silkworm (*Bombyx mori*) [11]. Sericin is characterized by the presence of 32% serine, which is the main amino acid of the natural moisture factor (NMF) in human skin; therefore, sericin has excellent moisturizing properties that may be helpful for treating hypohidrosis. Sericin also shows many biological activities and has been widely studied for potential use in medicines and biomaterials [12-16]. In 2009, Aramwit *et al*. showed that sericin significantly decreased the levels of the pro-inflammatory cytokines tumor necrosis factor-α (TNF-α) and interleukin-1β (IL-1β) in sericin-treated wounds in rats 7 days after an injury compared with the levels found in normal saline-soaked wounds and cream base-treated wounds [17]. As previously mentioned, the immune-inflammatory hypothesis considers UP a dermatologic manifestation of chronic inflammation and treats the condition as a possible result of derangements in the immune system that are based on a pro-inflammatory pattern. Based on this reasoning, sericin may help to relieve UP.

At present, there is no widely accepted treatment for UP. Topical products, such as moisturizers and emollients, are typically used to alleviate symptoms. Because sericin can suppress the release of pro-inflammatory cytokines, the present study was designed to investigate the short-term safety and efficacy of sericin cream for treating UP in ESRD patients. The quality of life of ESRD patients after using sericin cream was also evaluated.

## Results

### Molecular weight and amino acid composition of sericin

Sodium dodecyl sulfate polyacrylamide gel electrophoresis (SDS-PAGE) of sericin prepared by the high-temperature and high-pressure degumming technique showed continuous bands with molecular weights ranging from 50–150 kDa. The highest-intensity band had an apparent molecular weight of approximately 100 kDa. The amino acid composition of sericin extracted with this method is given in Table 1.

**Table 1:** Amino acid composition of sericin

**Amino acid**	**Amount (%)**
Serine (Ser)	33.63
Aspartic acid (Asp)	15.64
Glycine (Gly)	15.03
Threonine (Thr)	8.16
Glutamic acid (Glu)	4.61
Alanine (Ala)	4.10
Tyrosine (Tyr)	3.45
Methionine (Met)	3.39
Valine (Val)	2.88
Arginine (Arg)	2.87
Lysine (Lys)	2.35
Histidine (His)	1.06
Leucine (Leu)	1.00
Isoleucine (Ile)	0.56
Proline (Pro)	0.54
Cysteine (Cys)	0.44
Phenylalanine (Phe)	0.28

These mean values were obtained by triplicate analysis.

### Study population

Fifty hemodialysis patients with ESRD were enrolled in this study; however, only 47 subjects completed the treatment (3 patients were withdrawn due to relocation). Thirty of the 47 subjects were female (63.83%), and 17 were male (36.17%); the mean age was 49.6 ± 11.2 years. The average duration of dialysis was 24.6 ± 3.1 months. Table 2 shows the characteristics and biochemical parameters of the study population. Most of the biochemical parameters, such as calcium (9.87 ± 1.32 mg/dL), phosphorus (4.35 ± 1.02 mg/dL), albumin (3.96 ± 0.62 g/dL), total bilirubin (0.32 ± 0.11 mg/dL) and liver enzyme (alanine transaminase (ALT) 14.23 ± 5.98 IU/L, aspartate aminotransferase (AST) 15.32 ± 5.12 IU/L and alkaline phosphatase 96.58 ± 40.32 IU/L) levels, were within the normal range. The baseline characteristics of each parameter, including skin hydration, skin irritation and skin pigmentation on the right and left extremities, showed no significant differences (*p* = 0.819 and 0.982 for skin hydration on arms and legs, *p* = 0.892 and 0.857 for skin irritation on arms and legs, *p* = 0.834 and 0.901 for skin pigmentation on arms and legs, respectively). The average itching score of the subjects using the visual analogue scale (VAS) at baseline was 7.05, which was considered moderate to severe (VAS < 4.0 is considered to reflect mild pruritus, while VAS 4.0 - 6.9 indicates moderate pruritus, and VAS > 7.0 is considered to indicate severe pruritus [10]). At baseline, both the arms and legs of the subjects showed some degree of skin irritation. None of the subjects reported any allergy or dermatological symptoms caused by the sericin or the cream base. 

**Table 2:** Baseline characteristics of subjects (N = 47)

	**Characteristics**		
Gender (M:F)	17:30		
Age (years)	49.6 ± 11.2		
Dialysis duration (months)	24.6 ± 3.1		
Hematocrit (%)	30.12 ± 3.55		
BUN (mg/dL)	57.89 ± 12.65		
Creatinine (mg/dL)	10.98 ± 2.25		
Calcium (mg/dL)	9.87 ± 1.32		
Phosphorus (mg/dL)	4.35 ± 1.02		
Albumin (g/dL)	3.96 ± 0.62		
ALT (IU/L)	14.23 ± 5.98		
AST (IU/L)	15.32 ± 5.12		
Alkaline phosphatase (IU/L)	96.58 ± 40.32		
Total bilirubin (mg/dL)	0.32 ± 0.11		
Itching (scale 1–10)	7.05 ± 2.17		
**Skin parameters**	**Sericin cream treatment**	**Cream base treatment**	***p*-value**
*Skin hydration*			
Arm	28.67 ± 7.11	27.55 ± 7.84	0.819
	(13.90-44.90)	(13.36-44.34)	
Leg	25.10 ± 7.67	23.29 ± 7.37	0.982
	(13.98-49.70)	(7.74-38.88)	
*Skin irritation*			
Arm	409.34 ± 232.09	318.50 ± 484.26	0.892
	(177.60-1,441.00)	(217.00-2,558.40)	
Leg	364.74 ± 120.20	308.22 ± 116.14	0.857
	(177.00-685.40)	(158.80-818.20)	
*Skin pigmentation*			
Arm	373.56 ± 142.32	360.35 ± 249.29	0.834
	(155.60-729.80)	(145.80-1,400.00)	
Leg	435.94 ± 178.06	402.25 ± 154.53	0.901
	(199.00-1,182.80)	(196.00-811.00)	

BUN = blood urea nitrogen, ALT = alanine transaminase, AST = aspartate aminotransferase.

**Figure 1:**
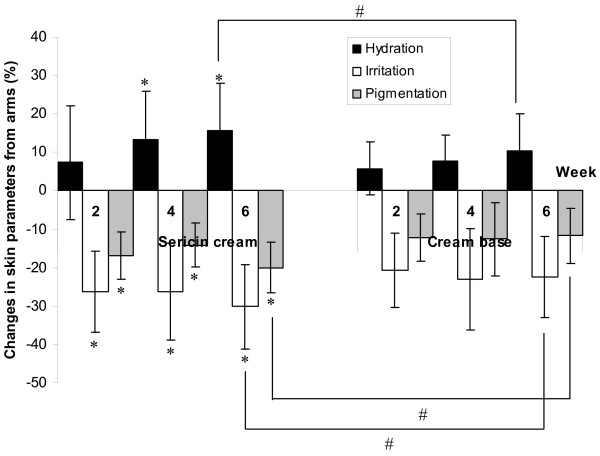
**Changes in skin parameters of hydration, irritation and pigmentation on the arms. **Detailed legend: The changes in the skin parameters of hydration (measured by Corneometer), irritation and pigmentation (measured by Mexameter) on patients’ arms treated with sericin cream or with cream base at weeks 2, 4 and 6 of treatment compared to baseline (* indicates significant differences compared to baseline; # indicates significant differences between treatments) (*p * < 0.05).

**Figure 2:**
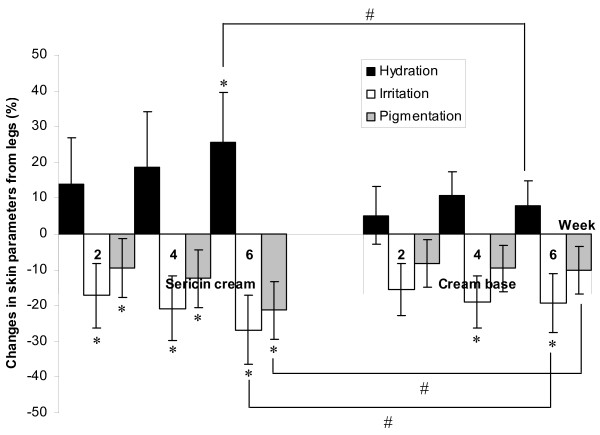
**Changes in skin parameters of hydration and irritation and pigmentation on the legs. **Detailed legend: The changes in the skin parameters of hydration (measured by Corneometer), irritation and pigmentation (measured by Mexameter) on patients’ legs treated with sericin cream or cream base at weeks 2, 4 and 6 of treatment compared to baseline (* indicates significant differences compared to baseline; # indicates significant differences between treatment) (*p * < 0.05).

The level of skin hydration in the patients’ extremities increased after treatment with either sericin or cream base. The same patients received sericin and placebo (cream base) treatment and that application of each compound were confined to one side of the body. On the sericin-treated side of the body, the skin hydration of the arms was 28.67 ± 7.11 at baseline, and it increased to 33.62 ± 6.93 after treatment for 6 weeks (*p* = 0.047), while the skin hydration of the legs, which was 25.10 ± 7.67 at baseline, increased to 29.05 ± 7.74 after 6 weeks of treatment (*p* = 0.025). On the cream-base-treated side of the body, the skin hydration of the arms was 27.55 ± 7.84 at baseline, and it increased to 29.40 ± 4.92 after treatment for 6 weeks (*p* = 0.593); the skin hydration of the legs, which was 23.29 ± 7.37 at baseline, changed to 26.02 ± 6.47 after 6 weeks of treatment (*p* = 0.276). The skin hydration changes were significantly higher on the side that received the sericin cream than on the side that received the cream base, and a significant difference between the sericin cream and cream base treatments was found in the level of skin hydration in both the arms and legs during the sixth week of treatment (*p* = 0.041 for arms and *p* = 0.022 for legs, respectively). Table 3 shows the skin parameters for hydration (measured by the Corneometer) and for irritation and pigmentation (measured by the Mexameter) of the subjects’ extremities at weeks 2, 4 and 6 after treatment. Figure 1 and 2 illustrate the percent changes in the parameters of the skin that received the sericin cream or the cream base during weeks 2–6 compared to the baseline. Six weeks after treatment, the average level of skin hydration on the side of the body that received the sericin cream was significantly increased compared to the baseline (*p* = 0.047 for arms and *p* = 0.025 for legs), while the side that received the cream base showed no significant difference (*p* = 0.593 for arms and *p* = 0.276 for legs) in skin hydration. The arms of the patients who were treated with the sericin cream showed significant differences in the level of skin hydration four weeks after the treatment compared to baseline (*p* = 0.022).

**Table 3:** Skin parameters of hydration, irritation and pigmentation on the subjects’ extremities after treatment

**Parameters**	**Baseline**		**2**^**nd**^**Week**		**4**^**th**^**Week**		**6 **^**th **^**Week**	
	**SS cream**	**Cream base**	**SS cream**	**Cream base**	**SS cream**	**Cream base**	**SS cream**	**Cream base**
*Hydration*								
Arm	28.67±7.11	27.55±7.84	29.59±6.07	28.00±5.73	32.41±7.10*	29.36±6.01	33.62±6.93*	29.40±4.92
Leg	25.10±7.67	23.29±7.37	25.72±5.74	24.16±5.95	27.24±6.23	26.43±5.90	29.05±7.74*	26.02±6.47
*Irritation*								
Arm	409.34±232.09	318.50±484.26	283.85±77.33*	302.17±74.87	276.53±66.67*	286.29±77.26	260.71±69.30*	291.24±82.68
Leg	364.74±120.20	308.22±116.14	295.59±75.21*	283.82±75.04	267.93±78.03*	255.42±74.78*	261.54±79.77*	256.46±69.06*
*Pigmentation*								
Arm	373.56±142.32	360.35±249.29	308.09±108.37*	342.73±130.47	302.62±111.02*	349.71±132.26	285.42±116.99*	338.88±115.54
Leg	435.94±178.06	402.25±154.53	374.51±131.43*	386.38±141.47	347.51±139.57*	378.88±115.54	320.72±142.50*	375.03±133.28

* indicates a significant difference compared with the same treatment at baseline (*p * < 0.05).

SS = silk sericin.

Skin irritation was measured based on the redness of the skin; both the sericin cream and the cream base reduced the level of skin irritation throughout the study period. As shown in Table 3, the arms and legs that were treated with sericin cream showed statistically significantly reduced irritation or redness from the second week after treatment until the end of the study compared to the baseline (*p* = 0.031 for arms and *p* = 0.040 for legs). The degree of redness of the legs of the patients who were treated with the cream base during weeks 4 (*p* = 0.048) and week 6 (*p* = 0.036) was also significantly reduced compared to the baseline. In addition, a significant difference between treatment with sericin cream and cream base was found in the level of skin irritation of both arms (*p* = 0.013) and legs (*p* = 0.027) after 6 weeks of treatment.

The use of sericin cream significantly reduced the darkness of the skin on both the arms and legs of the patients, as shown in Table 3. In addition, in the sixth week of treatment, there was a significant difference in the level of skin pigmentation in the arms and legs treated with the sericin cream compared to those treated with the cream base (Figure 1 and 2, *p* = 0.032 for arms and *p* = 0.021 for legs, respectively). The cream base showed no statistically significant effect on the level of skin pigmentation, and no significant difference compared to baseline was found in the skin pigmentation of the patients’ arms and legs after use of the cream base for 6 weeks (*p* = 0.082 for arms and *p* = 0.067 for legs, respectively).

**Figure 3:**
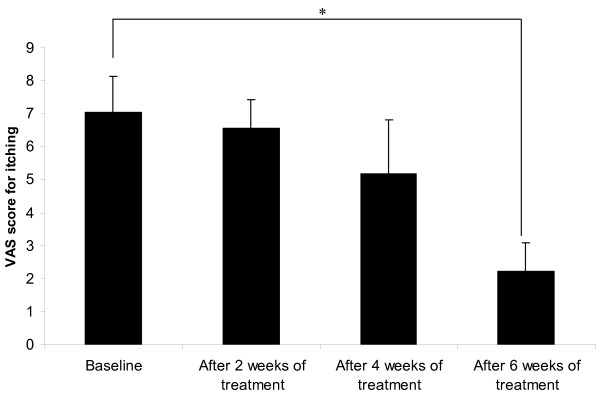
**The mean VAS score for itching. **Detailed legend: The mean VAS score for itching at baseline and after 2, 4 and 6 weeks of treatment (* indicates significant difference, *p * < 0.05).

The mean VAS scores for itching at baseline and after 2, 4 and 6 weeks of treatment are shown in Figure 3; the scores shown are based on the overall symptoms without differentiating between the sericin cream and cream base treatments. The mean pruritus score gradually decreased from the beginning of the study and with increasing weeks of treatment. The mean pruritus score at baseline was 7.05 ± 2.17 (range 1–10, median 8), which is indicates moderate to severe pruritus [10,18]; the mean score decreased to 2.23 ± 1.73 (range 0–6, median 2), indicating mild pruritus [10,18], after 6 weeks of treatment (*p* = 0.008). 

Table 4 shows the mean score assigned to each domain of the Kidney Disease Quality of Life Short Form (KDQOL-SF) by the hemodialysis patients on the enrollment day and after 6 weeks of treatment. As above, the quality-of-life score was based on the patients’ overall symptoms without differentiating between the sericin cream and cream base treatments. On the enrollment day, the mean scores ranged from 43.05 for general health to 83.84 for the domain related to dialysis staff encouragement. After 6 weeks of treatment, the mean scores ranged from 44.21 for kidney disease burden to 87.50 for encouragement by the dialysis staff. We found a better quality of life in all the measured domains, including sleep and mood/emotional distress, after the treatment period. When the mean score on the enrollment day was compared with the mean score on the day after completion of treatment, significant differences were found in some domains, including pain (*p* = 0.001), the symptoms/problems list in kidney disease (*p* = 0.000), the effect of kidney disease on daily life (*p* = 0.008) and sleep, the most relevant parameter for itching (*p* = 0.014). The overall score increased from 60.00 at the time of enrollment to 61.95 after 6 weeks of treatment, although this difference was not statistically significant (*p* = 0.091).

**Table 4:** Mean scores for each domain of the KDQOL-SF in hemodialysis patients on the enrollment day and after 6 weeks of treatment (N = 47)

	**Enrollment Day**	**After treatment for 6 weeks**
**Physical component score (PCS)**		
Physical functioning	73.90 ± 18.99	77.93 ± 18.57
Role limitations-physical	50.00 ± 42.20	51.83 ± 44.16
Pain	48.29 ± 16.96	74.27 ± 17.36*
General health	43.05 ± 24.08	47.32 ± 22.17
**Mental component score (MCS)**		
Energy/fatigue	50.73 ± 18.93	56.59 ± 17.62
Social function	69.82 ± 20.53	78.35 ± 22.54
Role limitations-emotional	56.10 ± 42.45	61.79 ± 41.87
Emotional well-being	61.17 ± 15.60	64.98 ± 16.51
**Kidney disease component score (KDCS)**		
Symptoms/problems list	64.33 ± 9.98	78.51 ± 10.44*
Effect of kidney disease on daily life	59.91 ± 20.46	68.22 ± 16.33*
Burden of kidney disease	43.45 ± 29.38	44.21 ± 25.29
Cognitive function	65.53 ± 18.25	69.27 ± 19.43
Work status	60.98 ± 41.10	68.29 ± 39.93
Sexual function	71.74 ± 33.12	75.00 ± 23.62
Quality of social interaction	70.08 ± 18.80	76.91 ± 16.06
Sleep	43.41 ± 22.08	52.74 ± 18.22*
Social support	70.33 ± 22.52	76.02 ± 25.01
Dialysis staff encouragement	83.84 ± 13.76	87.50 ± 12.18
Patient satisfaction	70.73 ± 20.34	78.05 ± 17.65
**Overall score**	60.00 ± 21.68	61.95 ± 19.90

* Indicates significant differences compared to baseline, *p * < 0.05.

## Discussion

This study has shown that the use of sericin cream can reduce UP in hemodialysis patients according to their VAS scores. Sericin cream can also significantly increase skin hydration and reduce skin irritation and skin pigmentation in patients. The molecular weight analysis of the sericin used in this study gave results that agree with those reported by Sprague, who indicated that sericin is a mixture of at least 15 different polypeptide chains ranging in size from 20–220 kDa [19]. Similar to the earlier report by Kato *et al*., serine was the most abundant amino acid found in sericin [20].

Although antihistamines, anti-allergic agents and topical corticosteroids are commonly used to treat UP in ESRD patients, their effectiveness is sometimes limited [2,8,21,22]. Alternative and effective treatments for intractable pruritus that generate low levels of adverse reactions, particularly in sensitive patients such as the ESRD group, must therefore be developed. This clinical study is the first to suggest that a sericin cream is beneficial for treating pruritus in hemodialysis patients. The benefits of this treatment apparently result from increases in skin hydration and suppression of pro-inflammatory cytokines, as shown in our earlier study [17,23], thereby resulting in less skin irritation without any allergic reactions.

Pruritus associated with chronic kidney disease has been shown to be associated with elevated levels of C-reactive protein and other inflammatory cytokines. This evidence suggests that there is an inflammatory component in this form of pruritus [24]. Some authors consider UP to be a skin manifestation of chronic inflammation [25]. Recently, the “persistent microinflammation” theory, in which it is suggested that inflammation may be related to the genesis of UP, was proposed [26]. The skin of ESRD patients with UP contains an increased number of mast cells, and these cells can release various substances such as histamine, interleukins (IL) and tumor necrosis factor (TNF) [27-30].

Pruritic skin can appear normal except for dryness; because the severity of pruritus is closely correlated with skin dryness [31], xerosis treatment normally begins with a topical agent such as a gentle moisturizing cream. Because sericin is well known for its moisturizing effect and its ability to reduce the generation of pro-inflammatory cytokines by fibroblasts, this study assessed the usefulness of sericin cream in treating UP. Evaluating the degree of pruritus was difficult because it is a subjective symptom; therefore, functional measurements of skin conditions known to be associated with pruritus, such as skin hydration and skin irritation, were considered to be objective indices. We found that both sericin cream and the cream base used to prepare the sericin cream increased the moisture content of the stratum corneum; however, the level of hydration was significantly higher in the skin treated with sericin cream. The side of the body treated with sericin cream had significantly lower levels of post-treatment dryness compared to pretreatment dryness and lower total dermatological severity scores in parameters such as skin pigmentation.

The pathogenesis of ESRD itself is associated with inflammation and oxidative stress, as reflected in studies of plasma biochemistry, including the levels of the cytokines IL and TNF-α [32-34], classic markers of inflammatory processes that are strongly affected by pathological conditions [35]. These cytokines are upregulated in the plasma of ESRD patients [32,36]. The skin inflammation associated with the release of IL and TNF-α is further complicated by the inflammatory lesions that occur secondary to scratching [37]. Our results indicate that improvement in skin hydration and suppression of the release of pro-inflammatory cytokines in skin might be the mechanism by which sericin improves UP. Skin irritation, which can be caused by either skin inflammation or by scratching, was substantially reduced in the sericin group compared with the cream base group after 6 weeks of treatment. This result confirmed that sericin reduces skin irritation in patients with UP and suggested that it might be due to suppression of the inflammatory cytokines in skin. Similar results have been obtained in animal studies [23].

Secondary skin lesions that can result from the itch-scratch cycle include excoriations, hyperpigmentation, lichenification, prurigo nodules and scars [38]. Chronic renal failure is usually accompanied by a variety of cutaneous manifestations; dermal manifestations, including hyperpigmentation, have also been reported, and these can exact a considerable toll on the quality of life [39-41]. Although skin pigmentation is not related to specific clinical symptoms, it has an important effect on patient satisfaction. Sericin has been reported to have activity against tyrosinase [15], an enzyme related to melanin production; as this study shows, treatment with sericin results in significant reduction in the skin color of patients.

The pruritus/mortality relationship may be strongly attributed to sleep disturbances, as previously mentioned in the DOPPS [8,9,42]. Many previous studies have found a strong association between inflammation, as measured by C-reactive protein or inflammatory cytokines, and sleep disturbances in dialysis patients [43-45]. The significant effects of pruritus on sleep, mood and social functioning require further investigation with the goal of improving the available treatments for this serious ESRD complication. We found that reduction in itching intensity from moderate to severe pruritus at the time of enrollment to mild pruritus after 6 weeks of treatment was associated with a better quality of life in all of the measured domains, including the mental, physical and kidney disease components. The KDQOL-SF score in the ESRD patients indicated improvements in several domains regarding the patients’ quality of life, particularly pain and sleep, which are relevant to itching. A better quality of sleep may reflect some degree of relief from itching achieved through the treatments.

This study has certain limitations. First, the study design was an in-subject controlled study; it cannot be determined whether the improvements in the itching evaluation and the quality of life were from the sample (sericin cream) or the placebo (cream base). Moreover, due to the in-subject controlled design, the biochemical parameters could not be evaluated at the end of the study. Second, the study included a small number of patients, and these were followed for a relatively short time. A long-term study with a larger sample size is necessary. In addition to studies of hemodialysis patients, similar studies of other ESRD patients, such as peritoneal dialysis and kidney transplant patients, are also necessary to confirm the findings presented here.

## Conclusions

Our study shows that sericin reduces pruritus in patients with UP. The use of sericin cream significantly increased the level of skin hydration after 6 weeks of treatment compared to baseline and to the use of the cream base. The use of sericin cream also significantly reduced the level of skin irritation and pigmentation after 6 weeks of treatment compared to baseline, while use of the cream base reduced skin pigmentation slightly but not significantly. The results of this study suggest that sericin cream may be a good choice for treating pruritus in hemodialysis patients.

## Methods

### Preparation of silk sericin cream

Because commercial sericin cream is unavailable, the sericin cream used in this study was prepared from raw materials. *Bombyx mori* cocoons were purchased from Chul Thai Silk Co., Ltd. (Petchaboon province, Thailand). The cocoons were extracted with purified water (1 g of dry silk cocoon: 30 mL of water) using a high temperature and pressure degumming technique in an autoclave (SS-320, Tomy Seiko Co., Ltd., Tokyo, Japan) at 121°C and 15 psi for 60 min. This technique has been shown to be safe for the preparation of material used on keratinocyte and fibroblast cells and can activate high collagen production related to wound healing [46]. After filtration through a membrane (Whatman filter paper No. 1, Whatman PLC, Kent, United Kingdom) to remove fibroin, sericin powder was obtained by freezing and lyophilizing the sericin solution with a Heto LL3000 lyophilizer (Allrod, Denmark). Petroleum jelly, mineral oil, lanolin, glycerin, bisabolol, triethanolamine stearate, propylparaben and methylparaben were used to formulate a cream base. For an 8% sericin cream, a concentration that has been shown to be safe and effective in the treatment of second-degree burn wounds, the sericin powder was dissolved in warm water and then mixed with the other ingredients during the cream-forming process.

### Molecular weight determination of sericin

To determine the molecular weight of sericin, SDS-PAGE was performed as previously described, with some modifications [47]. Briefly, samples were prepared for SDS-PAGE by adding an equal volume of sample buffer (0.25 M Tris–HCl, pH 7.0 containing 4% SDS, 10% sucrose, 10% 2-mercaptoethanol and 0.025% bromophenol blue) to each protein solution. Each sample was then incubated at 98°C for 2–3 min and loaded onto a 5%-20% gradient gel (Atto Corporation, Tokyo, Japan). Electrophoresis was performed in 125 mM Tris base with 0.96 M glycine and 0.5% SDS, and the polypeptide bands were detected using silver staining.

### Amino acid analysis of sericin

The amino acid composition of sericin was determined using an amino acid analyzer (Hitachi L-8500A, Tokyo, Japan). Samples for analysis were hydrolyzed in 4 M methanesulfonic acid containing 0.2% 3-(2-aminoethyl) indole (Wako Pure Chemical Industries, Ltd., Tokyo, Japan) at 100°C for 24 h under vacuum. The experiments were performed in triplicate.

### Study design

Skin hydration may be related to pruritus, and this parameter is sensitive to relative humidity, personal activities and diet. Therefore, an in-subject control using a split-body biometrological assessment (each patient received both treatments but on different sides of the body) was used to evaluate the safety and efficacy of the sericin cream for the treatment of UP in hemodialysis patients. Moreover, the distribution of pruritus between the patients was highly variable whereas the manifestation of mirror symmetry was an attribute they all shared [48]. An in-subject, randomized, double-blind, placebo-controlled experimental study was designed to investigate the effects of the sericin cream versus the cream base (placebo) in reducing the symptoms of UP (itching, dryness and redness) and skin pigmentation in stable maintenance hemodialysis patients. Each of the parameters, including skin hydration, skin irritation, skin pigmentation and itching score, was evaluated at baseline and at 2, 4 and 6 weeks after treatment intervention. The sericin cream and the cream base were identical in texture and scent. All of the products were packaged in containers that were label-free except for the treatment code number, and the packages were identical in shape, size and color; therefore, the treatment assignment remained unknown to the participants, the study investigators and the medical personnel. The subjects were recruited from December 2010 to February 2011, and the study was conducted between March 2011 and December 2011 at the Division of Nephrology, Phramongkutklao Hospital and at Priest Hospital, Thailand. Signed informed consent was obtained from all subjects after a thorough discussion of the protocol, its rationale and the potential risks. This study was approved by the Ethics Committee of the Institute Review Board at Phramongkutklao Hospital, Thailand and ended after the last participant completed the intervention.

### Study population

#### Inclusion criteria

All ESRD patients at Phramongkutklao and Priest Hospitals over 18 years of age who had received hemodialysis for at least 3 months were screened for this study. Having mild to severe pruritus as measured by the VAS during the previous 6 weeks was also an inclusion criterion. The patients were required to refrain from using any antipruritic treatment (oral or topical) for a period of not less than 2 weeks prior to the start of the study. Patients of both genders, regardless of comorbidities or prescribed medications, were eligible. Any medication that had an antipruritic effect was discontinued 2 weeks before the study. No changes in the patients’ prescription medications were required during this study with the exception of a concomitant antipruritic treatment.

#### Exclusion criteria

Pruritus caused by other skin diseases or medication was excluded by careful clinical assessment. Patients with a history of silk protein allergy, who were allergic to any compounds in the formula, or who had biliary atresia, liver problems, cancer, metabolic disorders or other diseases related to systemic pruritus were also excluded. Patients who had skin problems or rashes on their extremities (arms or legs) were also excluded from this study. Participants left the project when they could not comply with the treatment, when they were unwilling to continue with the study, or when the physician opined that other treatments were needed to relieve the symptoms. After reviewing patient profiles and explaining the protocol, 27 patients were excluded due to liver problems (N = 4), cancer (N = 3), metabolic disorder (N = 7), rashes on their extremities (N = 6) and refusal to participate in the study (N = 7). The remaining patients (N = 50) were enrolled in the study.

### Study treatment

In 2004, Okada and Matsumoto [49] evaluated the effect of an emollient containing a high water content on mild uremic pruritus; based on this study, the number of samples needed for a dependent sample was approximately 50 subjects. Split-body biometrological assessments were performed. The physician investigator enrolled the subjects into this study, and using a computer-generated block of four, another investigator generated the random allocation sequence that divided the patients into two groups. The identities of the patients in each group were concealed from both the investigators and the patients. The on-duty nurses assigned the participants to the intervention. The participants, investigators and those assessing the outcomes were blinded after assignment to the interventions. The patients in the first group received the sericin cream on their left extremities (left arm and left leg), while the other side of the body received the cream base. The patients in the second group received both the sericin cream and the cream base, but on opposite sides of the body from the first group. All of the patients were shown how to topically apply the assigned treatment evenly over the area indicated twice daily for a period of 6 weeks after showering.

### Measurement and outcome

The level of skin hydration on the arms and legs was assessed using a Corneometer® (KOKO Kosmetikvertrieb GmbH & Co., Leichlingen, Deutschland). The Corneometer® registers the moisture content in the surface layers of the skin as deep as 10–20 μm; the presence of capillary blood vessels and superficial skin fat do not influence this measurement. Skin irritation or erythema (measured by the redness of the skin) and skin pigmentation (measured by the melanin content) were assessed using a Mexameter linked to a Skin Diagnostic SD27 (Courage + Khazaka electronic GmbH, Köln, Germany). The measurement of melanin and the erythema readings are based on a light source with three specific wavelengths; the radiation is absorbed by the skin and reflected diffusely. A photodetector was used to analyze the diffuse reflection from the skin. The same measuring probe is used to quantify the skin redness (erythema) and to determine the skin pigmentation or the degree of skin darkness (melanin). The irritating effects of substances and the soothing effects of active agents can also be recorded by the investigator. Skin hydration, irritation and pigmentation values have no units because they are computer- generated based on the different dielectric constants of water (80.10 at 20°C) and other substances (typically < 7). The measuring capacitor shows changes of capacitance according to the moisture content of the samples (for hydration) and skin color (for irritation and pigmentation). Each parameter was measured at least three times in the same randomized area at patients’ extremities, and the mean value was used for the analysis. During the study, the patients were advised to consume similar types and amounts of food and beverages. Activities such as longer exposure to the sunlight and traveling were to be avoided to reduce any confounding factors. The percent changes in each parameter were calculated by subtracting the baseline score from the post-treatment scores at weeks 2, 4 and 6 according to the following equation:

(1) $$\%\text{changes in each parameter}=\left[\left(P_{t}-P_{0}\right)/P_{0}\right]\times 100\text{,}$$

where P_0_ is the value of each parameter at baseline (at the time of enrollment) and P_t_ is the value of each parameter during the follow-up period (2, 4 or 6 weeks). All of the measurements were performed in triplicate.

Because itching is a systemic symptom and likely to be generalized, most patients could not identify whether the itching occurred primarily on the right or left side of the body; therefore, itching was scored as an overall symptom. The severity of itching was systemically assessed on both the arms and legs of all patients using VAS on the enrollment day and every 2 weeks after treatment began. We used a VAS that consisted of a 10-cm horizontal line with no scale markings. The patients were asked to mark the intensity of their itching on the scale, with the strongest possible level of itching or unbearable pruritus marked on the right end of the line (10 cm) and no itching marked on the left end (0 cm) [50].

### Patients’ quality of life

The patients’ quality of life was assessed using the Thai version of the KDQOL-SF Version 1.3 [51]. Quality of life was evaluated on the enrollment day and after 6 weeks of treatment. The mean scores for the individual domain scores and for the three composite summary scores, which include the mental component score (MCS), the physical component score (PCS) and the kidney disease component score (KDCS), were compared as shown in Table 4. For the Hayes algorithm [52], the raw data obtained from the patients were first transformed into a pre-coded numeric value of 0–100; a higher transformed score reflected a better quality of life [51,53].

### Safety monitoring

The occurrence of allergic reactions during treatment with sericin cream was regularly evaluated by two dermatologists during each visit. The Naranjo algorithm was used to determine the likelihood of whether an adverse drug reaction was actually caused by the sericin cream or by other factors.

### Statistical analysis

The results are expressed as the mean ± SD unless otherwise indicated. Statistical analysis was performed using SPSS version 10.0 (SPSS Inc., Chicago, Illinois, USA). A bidirectional α-level of significance was set at *p* = 0.05 for all of the measurements. From the baseline to weeks 2, 4 and 6, the VAS score changes and the levels of skin hydration, irritation and pigmentation were computed for each patient within the treatment group using a repeated measure analysis of variance (ANOVA). The paired t-test was used to analyze changes in the patients’ quality of life between baseline and 6 weeks after treatment. The differences in each parameter for the patients receiving the sericin cream and the cream base were compared at each time point using Student’s t-test.

## Competing interests

The authors declare that they have no competing interests.

## Authors’ contributions

PA had full access to all of the data in the study, accepts responsibility for the integrity of the data and affirms that everyone who contributed significantly to the work has been listed. PA and OS developed the clinical study design and the experimental design. OK, TS and NB recruited the subjects and conducted the skin evaluation, patient consent, analysis and data interpretation. PA drafted the manuscript. All authors read the manuscript, provided critical input and approved the final version.

## Pre-publication history

The pre-publication history for this paper can be accessed here:

http://www.biomedcentral.com/1471-2369/13/119/prepub

## References

[B1] Feramisco JD Berger TG Steinhoff M Innovative management of pruritus Dermatol Clin 2010 28 3 467 478 10.1016/j.det.2010.03.004 20510757

[B2] Patel TS Freedman BI Yosipovitch G An update on pruritus associated with CKD Am J Kidney Dis 2007 50 1 11 20 10.1053/j.ajkd.2007.03.010 17591521

[B3] Mettang M Weisshaar E Pruritus: control of itch in patients undergoing dialysis Skin Therapy Lett 2010 15 2 1 5 20361169

[B4] Chen YC Chiu WT Wu MS Therapeutic effect of topical gamma-linolenic acid on refractory uremic pruritus Am J Kidney Dis 2006 48 1 69 76 10.1053/j.ajkd.2006.03.082 16797388

[B5] Urbonas A Schwartz RA Szepietowski JC Uremic pruritus–an update Am J Nephrol 2001 21 5 343 350 10.1159/000046272 11684792

[B6] Virga G Visentin I La Milia V Bonadonna A Inflammation and pruritus in haemodialysis patients Nephrol Dial Transplant 2002 17 12 2164 2169 10.1093/ndt/17.12.2164 12454228

[B7] Akhyani M Ganji MR Samadi N Khamesan B Daneshpazhooh M Pruritus in hemodialysis patients BMC Dermatol 2005 5 7 10.1186/1471-5945-5-7 15975150 PMC1184066

[B8] Pisoni RL Wikstrom B Elder SJ Akizawa T Asano Y Keen ML Saran R Mendelssohn DC Young EW Port FK Pruritus in haemodialysis patients: International results from the Dialysis Outcomes and Practice Patterns Study (DOPPS) Nephrol Dial Transplant 2006 21 12 3495 3505 10.1093/ndt/gfl461 16968725

[B9] Wikstrom B Itchy skin–a clinical problem for haemodialysis patients Nephrol Dial Transplant 2007 22 Suppl 5 v3 7 17586843 10.1093/ndt/gfm292

[B10] Narita I Alchi B Omori K Sato F Ajiro J Saga D Kondo D Skatsume M Maruyama S Kazama JJ Etiology and prognostic significance of severe uremic pruritus in chronic hemodialysis patients Kidney Int 2006 69 9 1626 1632 10.1038/sj.ki.5000251 16672924

[B11] Zhang YQ Applications of natural silk protein sericin in biomaterials Biotechnol Adv 2002 20 2 91 100 10.1016/S0734-9750(02)00003-4 14538058

[B12] Aramwit P Sangcakul A The effects of sericin cream on wound healing in rats Biosci Biotechnol Biochem 2007 71 10 2473 2477 10.1271/bbb.70243 17928707

[B13] Zhaorigetu S Yanaka N Sasaki M Watanabe H Kato N Inhibitory effects of silk protein, sericin on UVB-induced acute damage and tumor promotion by reducing oxidative stress in the skin of hairless mouse J Photochem Photobiol B 2003 71 1–3 11 17 14705634 10.1016/s1011-1344(03)00092-7

[B14] Zhaorigetu S Yanaka N Sasaki M Watanabe H Kato N Silk protein, sericin, suppresses DMBA-TPA-induced mouse skin tumorigenesis by reducing oxidative stress, inflammatory responses and endogenous tumor promoter TNF-alpha Oncol Rep 2003 10 3 537 543 12684620

[B15] Aramwit P Damrongsakkul S Kanokpanont S Srichana T Properties and antityrosinase activity of sericin from various extraction methods Biotechnol Appl Biochem 2010 55 2 91 98 10.1042/BA20090186 20055756

[B16] Aramwit P Siritientong T Kanokpanont S Srichana T Formulation and characterization of silk sericin-PVA scaffold crosslinked with genipin Int J Biol Macromol 2010 47 5 668 675 10.1016/j.ijbiomac.2010.08.015 20804781

[B17] Aramwit P Kanokpanont S De-Eknamkul W Srichana T Monitoring of inflammatory mediators induced by silk sericin J Biosci Bioeng 2009 107 5 556 561 10.1016/j.jbiosc.2008.12.012 19393558

[B18] Gupta MA Gupta AK Kirkby S Weiner HK Mace TM Schork NJ Johnson EH Ellis CN Voorhees JJ Pruritus in psoriasis. A prospective study of some psychiatric and dermatologic correlates Arch Dermatol 1988 124 7 1052 1057 10.1001/archderm.1988.01670070040016 3389849

[B19] Sprague KU The Bombyx mori silk proteins: characterization of large polypeptides Biochemistry 1975 14 5 925 931 10.1021/bi00676a008 1125178

[B20] Kato N Sato S Yamanaka A Yamada H Fuwa N Nomura M Silk protein, sericin, inhibits lipid peroxidation and tyrosinase activity Biosci Biotechnol Biochem 1998 62 1 145 147 10.1271/bbb.62.145 9501526

[B21] Schwartz IF Iaina A Uraemic pruritus Nephrol Dial Transplant 1999 14 4 834 839 10.1093/ndt/14.4.834 10328453

[B22] Mettang T Pauli-Magnus C Alscher DM Uraemic pruritus–new perspectives and insights from recent trials Nephrol Dial Transplant 2002 17 9 1558 1563 10.1093/ndt/17.9.1558 12198205

[B23] Aramwit P Kanokpanont S Punyarit P Srichana T Effectiveness and Inflammatory Cytokines Induced by Sericin Compared to Sericin in Combination with Silver Sulfadiazine Cream on Wound Healing Wounds 2009 21 8 198 206 25903672

[B24] Berger TG Steinhoff M Pruritus and renal failure Semin Cutan Med Surg 2011 30 2 99 100 10.1016/j.sder.2011.04.005 21767770 PMC3692272

[B25] Duque MI Thevarajah S Chan YH Tuttle AB Freedman BI Yosipovitch G Uremic pruritus is associated with higher kt/V and serum calcium concentration Clin Nephrol 2006 66 3 184 191 16995341 10.5414/cnp66184

[B26] Kimmel M Alscher DM Dunst R Braun N Machleidt C Kiefer T Stulten C van der Kuip H Pauli-Magnus C Raub U The role of micro-inflammation in the pathogenesis of uraemic pruritus in haemodialysis patients Nephrol Dial Transplant 2006 21 3 749 755 10.1093/ndt/gfi204 16249205

[B27] Lugon JR Uremic pruritus: a review Hemodial Int 2005 9 2 180 188 10.1111/j.1492-7535.2005.01130.x 16191067

[B28] Dugas-Breit S Schopf P Dugas M Schiffl H Rueff F Przybilla B Baseline serum levels of mast cell tryptase are raised in hemodialysis patients and associated with severity of pruritus J Dtsch Dermatol Ges 2005 3 5 343 347 10.1111/j.1610-0387.2005.05706.x 16372800

[B29] Szepietowski J Thepen T van Vloten WA Szepietowski T Bihari IC Pruritus and mast cell proliferation in the skin of haemodialysis patients Inflamm Res 1995 44 Suppl 1 S84 85 8521016 10.1007/BF01674408

[B30] Matsumoto M Ichimaru K Horie A Pruritus and mast cell proliferation of the skin in end stage renal failure Clin Nephrol 1985 23 6 285 288 4028525

[B31] Young AW Jr Sweeney EW David DS Cheigh J Hochgelerenl EL Sakai S Stenzel KH Rubin AL Dermatologic evaluation of pruritus in patients on hemodialysis N Y State J Med 1973 73 22 2670 2674 4519082

[B32] Pereira BJ Shapiro L King AJ Falagas ME Strom JA Dinarello CA Plasma levels of IL-1 beta, TNF alpha and their specific inhibitors in undialyzed chronic renal failure, CAPD and hemodialysis patients Kidney Int 1994 45 3 890 896 10.1038/ki.1994.117 8196293

[B33] Stenvinkel P Ketteler M Johnson RJ Lindholm B Pecoits-Filho R Riella M Heimburger O Cederholm T Girndt M IL-10, IL-6, and TNF-alpha: central factors in the altered cytokine network of uremia–the good, the bad, and the ugly Kidney Int 2005 67 4 1216 1233 10.1111/j.1523-1755.2005.00200.x 15780075

[B34] Bolton CH Downs LG Victory JG Dwight JF Tomson CR Mackness MI Pinkney JH Endothelial dysfunction in chronic renal failure: roles of lipoprotein oxidation and pro-inflammatory cytokines Nephrol Dial Transplant 2001 16 6 1189 1197 10.1093/ndt/16.6.1189 11390719

[B35] Portugal-Cohen M Oron M Ma'or Z Boaz M Shtendik L Biro A Cernes R Barnea Z Kazir Z Kohen R Noninvasive skin measurements to monitor chronic renal failure pathogenesis Biomed Pharmacother 2011 65 4 280 285 10.1016/j.biopha.2011.02.001 21549551

[B36] Khan SB Cook HT Bhangal G Smith J Tam FW Pusey CD Antibody blockade of TNF-alpha reduces inflammation and scarring in experimental crescentic glomerulonephritis Kidney Int 2005 67 5 1812 1820 10.1111/j.1523-1755.2005.00279.x 15840028

[B37] Ponticelli C Bencini PL Uremic pruritus: a review Nephron 1992 60 1 1 5 10.1159/000186696 1738396

[B38] Wang H Yosipovitch G New insights into the pathophysiology and treatment of chronic itch in patients with end-stage renal disease, chronic liver disease, and lymphoma Int J Dermatol 2010 49 1 1 11 10.1111/j.1365-4632.2009.04249.x 20465602 PMC2871329

[B39] Bencini PL Montagnino G Citterio A Graziani G Crosti C Ponticelli C Cutaneous abnormalities in uremic patients Nephron 1985 40 3 316 321 10.1159/000183485 4010846

[B40] Avermaete A Altmeyer P Bacharach-Buhles M Skin changes in dialysis patients: a review Nephrol Dial Transplant 2001 16 12 2293 2296 10.1093/ndt/16.12.2293 11733617

[B41] Dyachenko P Shustak A Rozenman D Hemodialysis-related pruritus and associated cutaneous manifestations Int J Dermatol 2006 45 6 664 667 10.1111/j.1365-4632.2005.02592.x 16796623

[B42] Chiu YL Chen HY Chuang YF Hsu SP Lai CF Pai MF Yang SY Peng YS Association of uraemic pruritus with inflammation and hepatitis infection in haemodialysis patients Nephrol Dial Transplant 2008 23 11 3685 3689 10.1093/ndt/gfn303 18515654

[B43] Yang JY Huang JW Chiang CK Pan CC Wu KD Tsai TJ Chen WY Higher plasma interleukin-18 levels associated with poor quality of sleep in peritoneal dialysis patients Nephrol Dial Transplant 2007 22 12 3606 3609 10.1093/ndt/gfm231 17890740

[B44] Chiu YL Chuang YF Fang KC Liu SK Chen HY Yang JY Pai MF Peng YS Wu KD Tsai TJ Higher systemic inflammation is associated with poorer sleep quality in stable haemodialysis patients Nephrol Dial Transplant 2009 24 1 247 251 18664587 10.1093/ndt/gfn439

[B45] Chen HY Chiang CK Wang HH Hung KY Lee YJ Peng YS Wu KD Tsai TJ Cognitive-behavioral therapy for sleep disturbance in patients undergoing peritoneal dialysis: a pilot randomized controlled trial Am J Kidney Dis 2008 52 2 314 323 10.1053/j.ajkd.2008.03.012 18511165

[B46] Aramwit P Kanokpanont S Nakpheng T Srichana T The effect of sericin from various extraction methods on cell viability and collagen production Int J Mol Sci 2010 11 5 2200 2211 10.3390/ijms11052200 20559510 PMC2885102

[B47] Takasu Y Yamada H Tsubouchi K Isolation of three main sericin components from the cocoon of the silkworm, Bombyx mori Biosci Biotechnol Biochem 2002 66 12 2715 2718 10.1271/bbb.66.2715 12596874

[B48] Mathur VS Lindberg J Germain M Block G Tumlin J Smith M Grewal M McGuire D A longitudinal study of uremic pruritus in hemodialysis patients Clin J Am Soc Nephrol 2010 5 8 1410 1419 10.2215/CJN.00100110 20558560 PMC2924419

[B49] Okada K Matsumoto K Effect of skin care with an emollient containing a high water content on mild uremic pruritus Ther Apher Dial 2004 8 5 419 422 10.1111/j.1526-0968.2004.00175.x 15663539

[B50] Wahlgren CF Ekblom A Hagermark O Some aspects of the experimental induction and measurement of itch Acta Derm Venereol 1989 69 3 185 189 2566219

[B51] Homjean K Sakthong P Translation and cognitive testing of the Thai version of the kidney disease quality of life short-form questionnaires version 1.3 Thai J Pharm Pract 2010 2 1 3 14

[B52] Park HJ Kim S Yong JS Han SS Yang DH Meguro M Han CW Kohzuki M Reliability and validity of the Korean version of Kidney Disease Quality of Life instrument (KDQOL-SF) Tohoku J Exp Med 2007 211 4 321 329 10.1620/tjem.211.321 17409671

[B53] Al Jumaih A Al-Onazi K Binsalih S Hejaili F Al-Sayyari A A Study of Quality of Life and its Determinants among Hemodialysis Patients Using the KDQOL-SF Instrument in One Center in Saudi Arabia Arab J Nephrol Transplant 2011 4 3 125 130 22026335 10.4314/ajnt.v4i3.71024

